# Supplementary material for: Major vault protein suppresses obesity and atherosclerosis through inhibiting IKK–NF-κB signaling mediated inflammation
Source: Nat Commun. 2019 Apr 17;10:1801. doi: 10.1038/s41467-019-09588-x (PMC6470148; doi:10.1038/s41467-019-09588-x)
Supplement: Supplementary file 1 — Supplementary Information [file 41467_2019_9588_MOESM1_ESM.pdf]

Major vault protein suppresses obesity and atherosclerosis through inhibiting IKK-NF-κB signaling mediated inflammation

Ben *et al*.

Supplementary Figure 1.

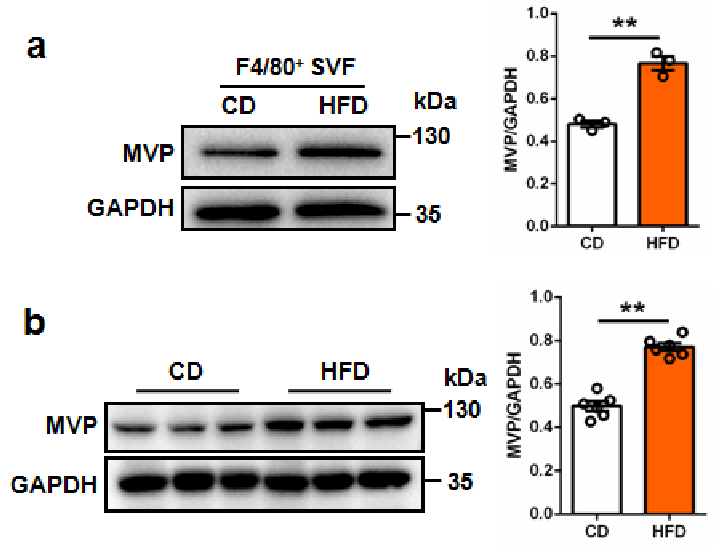

Supplementary Figure 1. Female C57BL/6J mice were fed a CD or a HFD for 7 weeks. **(a)** Western blot analysis of MVP expression in F4/80$^+$ macrophages isolated from gonadal white adipose tissue (gonWAT) SVFs (n = 3). **(b)** Western blot analysis of MVP in PMs (n = 6). Data are expressed as mean ± SEM. **P < 0.01 by Student's *t* test.

Supplementary Figure 2.

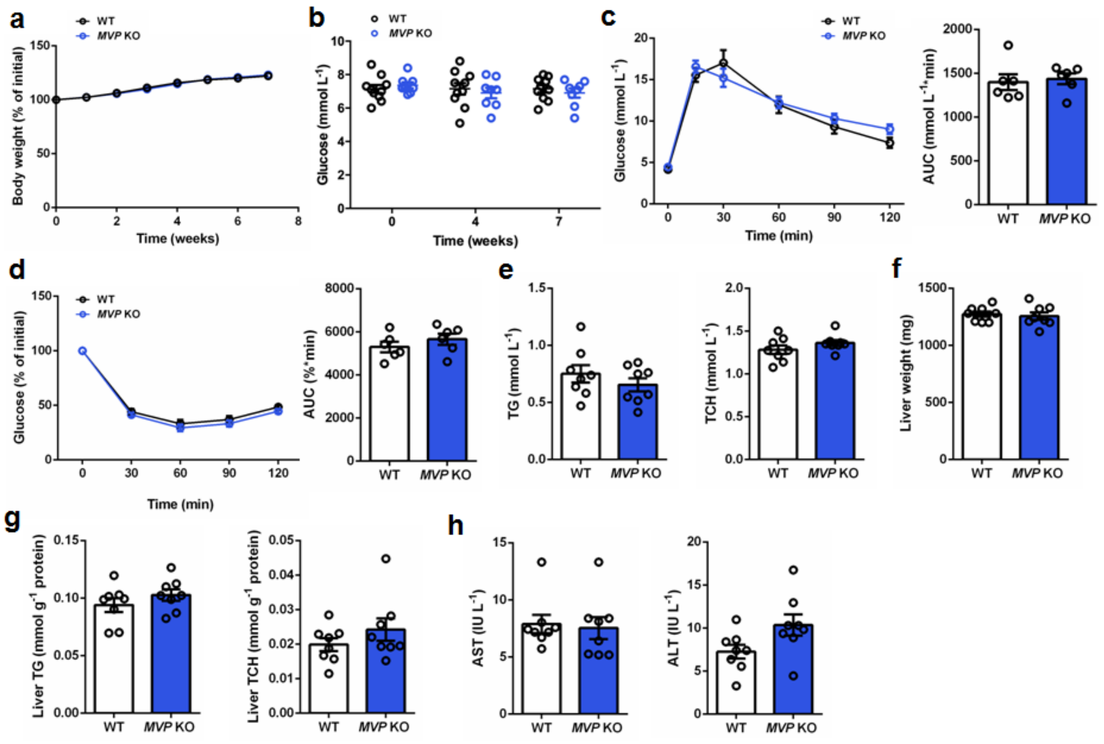

Supplementary Figure 2. Glycolipid metabolism in *MVP* KO male mice fed a CD.

WT and *MVP* KO male mice were fed a CD for 7 weeks. **(a)** The percentage of body weight gain in WT (n = 10) and *MVP* KO (n = 8) mice. **(b)** Fasting blood glucose level in WT (n = 10) and *MVP* KO (n = 8) mice. **(c-d)** GTT and ITT in WT and *MVP* KO mice (n = 6). **(e)** Plasma levels of TG and TCH in WT and *MVP* KO mice (n = 8). **(f-g)** Murine liver tissues were retrieved after 7 weeks of CD-feeding and their wet weight (f), TG and TCH levels (g) were determined (n = 8). (**h**) Plasma levels of AST and ALT in mice (n = 8). Data are expressed as mean ± SEM and analyzed by Student's *t* test.

Supplementary Figure 3.

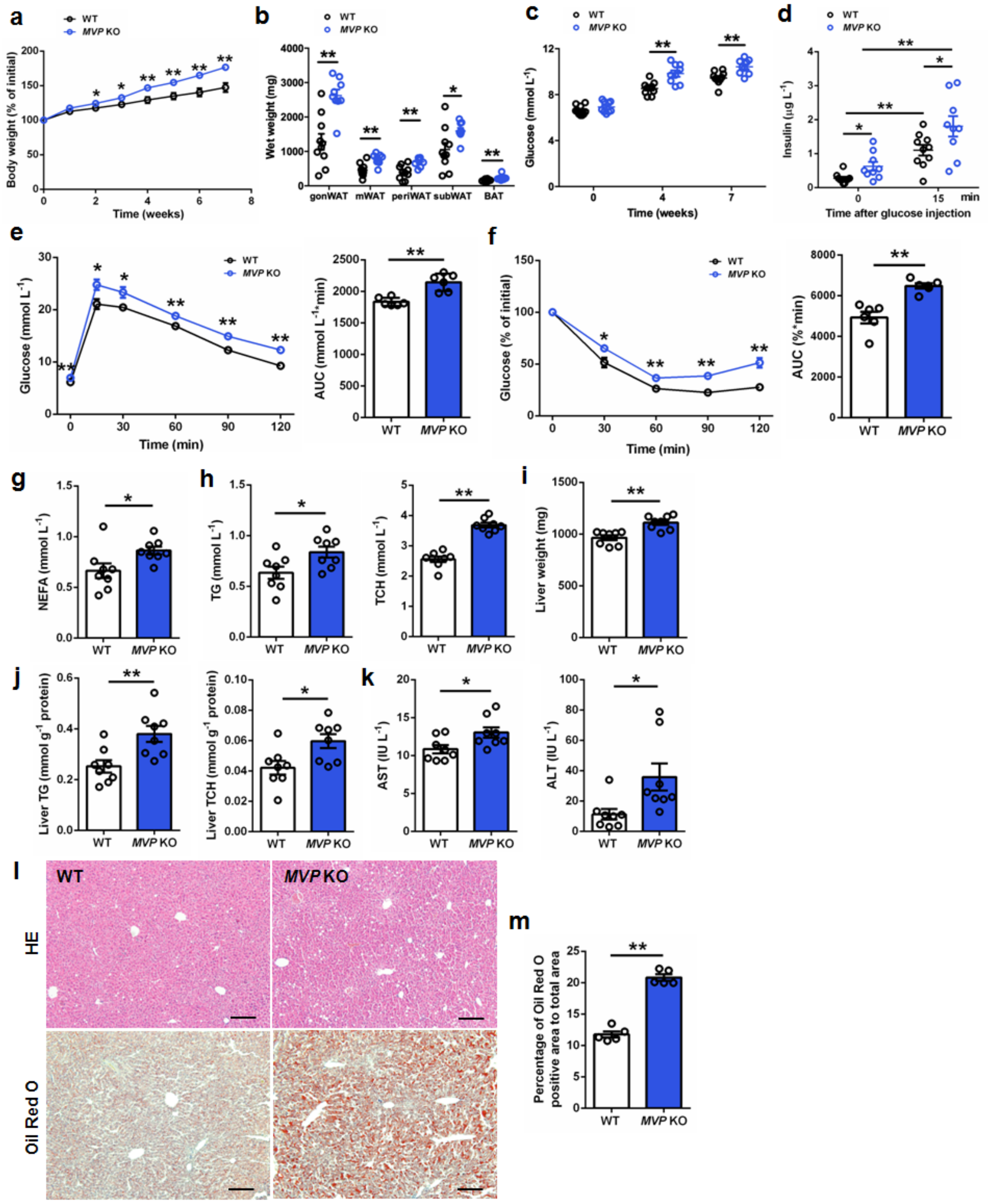

Supplementary Figure 3. MVP deficiency deteriorates HFD-induced metabolic disorders in female mice.

WT and *MVP* KO female mice were fed a HFD for 7 weeks. **(a)** The percentage of body weight gain in WT (n = 10) and *MVP* KO mice (n = 9). **(b)** Depot mass of gonadal (gon), mesentery (m), perirenal (peri), subcutaneous (sub) WAT and BAT in

WT (n = 10) and *MVP* KO mice (n = 9). **(c)** Fasting blood glucose in WT (n = 10) and *MVP* KO mice (n = 9). **(d)** Basal- and stimulated-insulin levels in WT (n = 10) and *MVP* KO mice (n = 9). **(e-f)** GTT and ITT in WT and *MVP* KO mice (n = 6). **(g-h)** Plasma levels of NEFA (g), TG and TCH (h) in WT and *MVP* KO mice (n = 8). **(i-j)** Murine liver tissues were retrieved after 7 weeks of HFD feeding and their wet weights (i), liver TG and TCH (j) levels were determined (n = 8). **(k)** Plasma levels of AST and ALT in mice (n = 8). **(l)** H&E (top) and Oil Red O (bottom) staining of representative liver sections obtained from HFD-fed WT (left) and *MVP* KO (right) mice. Scale bars, 100 μm. **(m)** Quantification of Oil Red O stained area of liver (n = 5). Data are expressed as mean ± SEM. *P < 0.05 and **P < 0.01 by Student's *t* test or ANOVA with post hoc test.

Supplementary Figure 4.

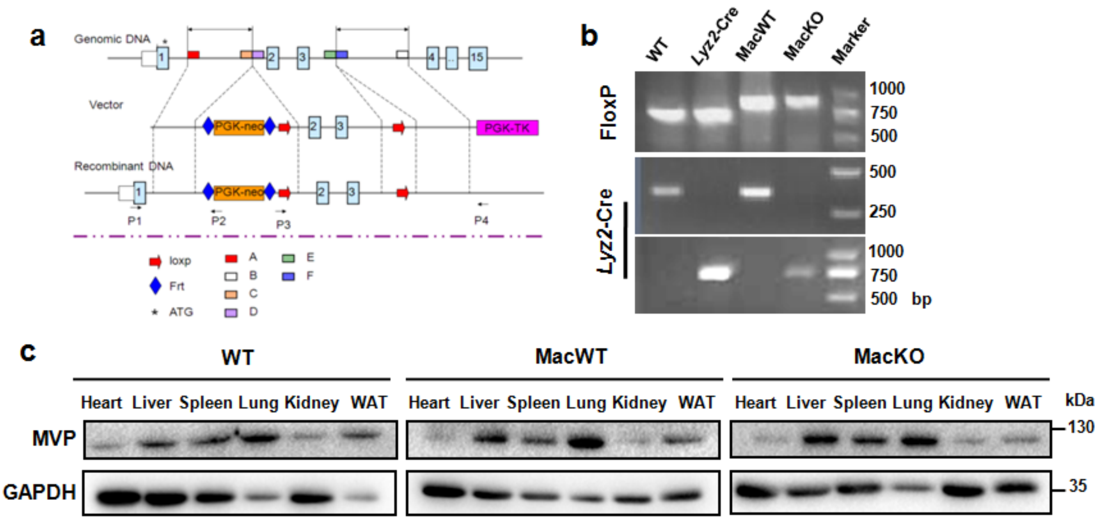

Supplementary Figure 4. Establishment of myeloid-specific *MVP* deficiency mice.

(a) Schematic work flow of the establishment of *MVP*$^{loxp/loxp}$ mouse strain. **(b)** Representative genotyping PCR amplification of WT, *Lyz2*-Cre, MacWT (*MVP*$^{loxp/loxp}$) and MacKO (*MVP*$^{loxp/loxp}$*Lyz2*-Cre) mice. **(c)** Representative western blot analysis of MVP in the indicated tissues from WT, MacWT and MacKO mice.

Supplementary Figure 5.

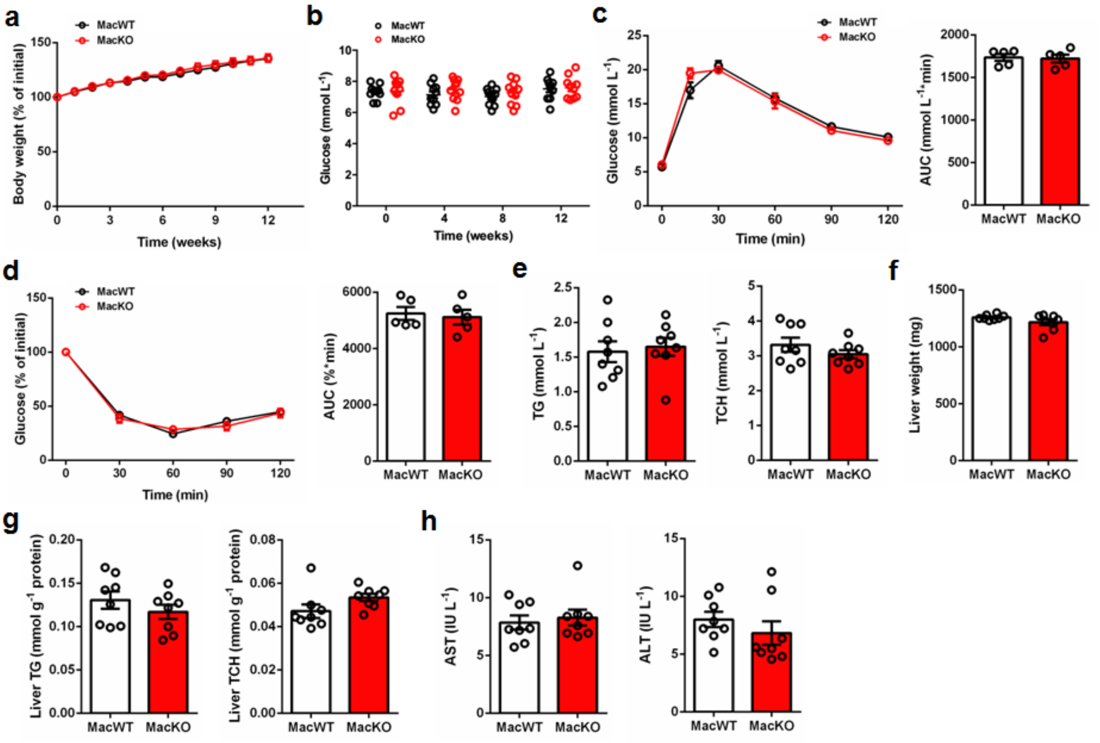

Supplementary Figure 5. Glycolipid metabolism in MacKO male mice fed a CD.

MacWT and MacKO male mice were fed a CD for 12 weeks. **(a)** The percentage of body weight gain in MacWT and MacKO mice (n = 10). **(b)** Fasting blood glucose level in MacWT and MacKO mice (n = 10). **(c-d)** GTT and ITT in MacWT and MacKO mice (n = 5). **(e)** Plasma levels of TG and TCH in MacWT and MacKO mice (n = 8). **(f-g)** Murine liver tissues were retrieved after 12 weeks of CD feeding and their wet weight (f), liver TG and TCH levels (g) were determined (n = 8). **(h)** Plasma levels of AST and ALT in mice (n = 8). Data are expressed as mean ± SEM and analyzed by Student's *t* test.

Supplementary Figure 6.

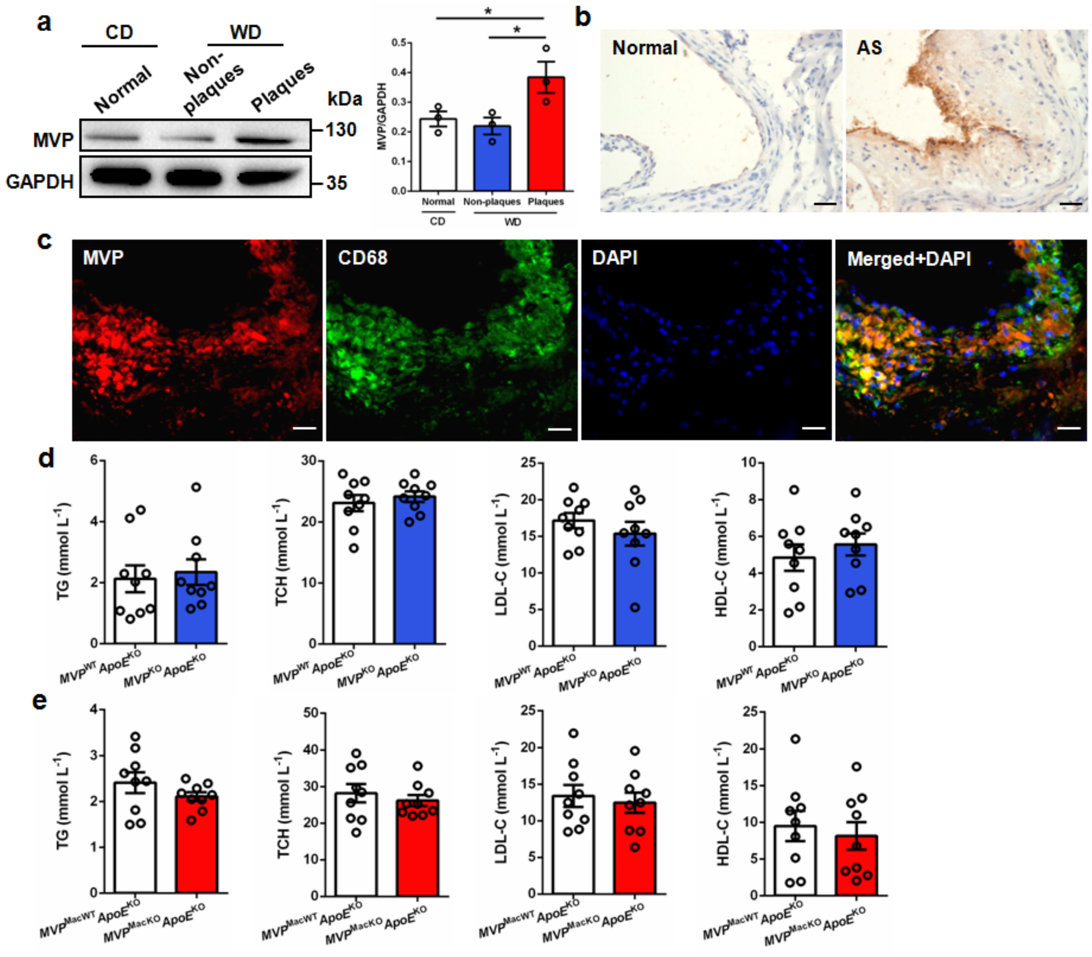

Supplementary Figure 6. *ApoE*^{KO} mice were fed a CD or a WD for 10 weeks. **(a)** Western blot analysis of MVP expression in murine aortas (n = 3). **(b)** IHC staining of MVP in aortic root sections of *ApoE*^{KO} mice. Scale bars, 20 µm. **(c)** Immunofluorescence staining of MVP (red), CD68 (green) and their co-localization (yellow merge) in atherosclerotic plaques. Nuclei were stained with DAPI (blue). Scale bars, 20 µm. **(d)** Lipids profiles in *MVP*^{KO}*ApoE*^{KO} and *MVP*^{WT}*ApoE*^{KO} mice after 10 weeks of WD feeding (n = 9). **(e)** Lipids profiles in *MVP*^{MacKO}*ApoE*^{KO} and *MVP*^{MacWT}*ApoE*^{KO} mice after 12 weeks of WD feeding (n = 9). Data are expressed as mean ± SEM. *P < 0.05 by Student's *t* test or ANOVA with post hoc test.

Supplementary Figure 7.

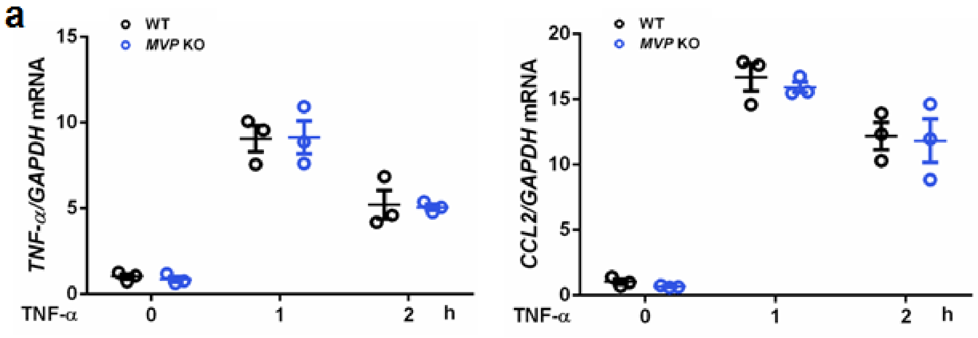

Supplementary Figure 7. (a) PMs isolated from WT and *MVP* KO CD-fed mice were treated with TNF-α (10 ng ml^{-1}) for indicated times. mRNA levels of inflammatory mediators (*TNF-α* and *CCL2*) were assessed by RT-qPCR (n = 3). Representative results from three independent experiments are shown. Data are expressed as mean ± SEM.

Supplementary Figure 8.

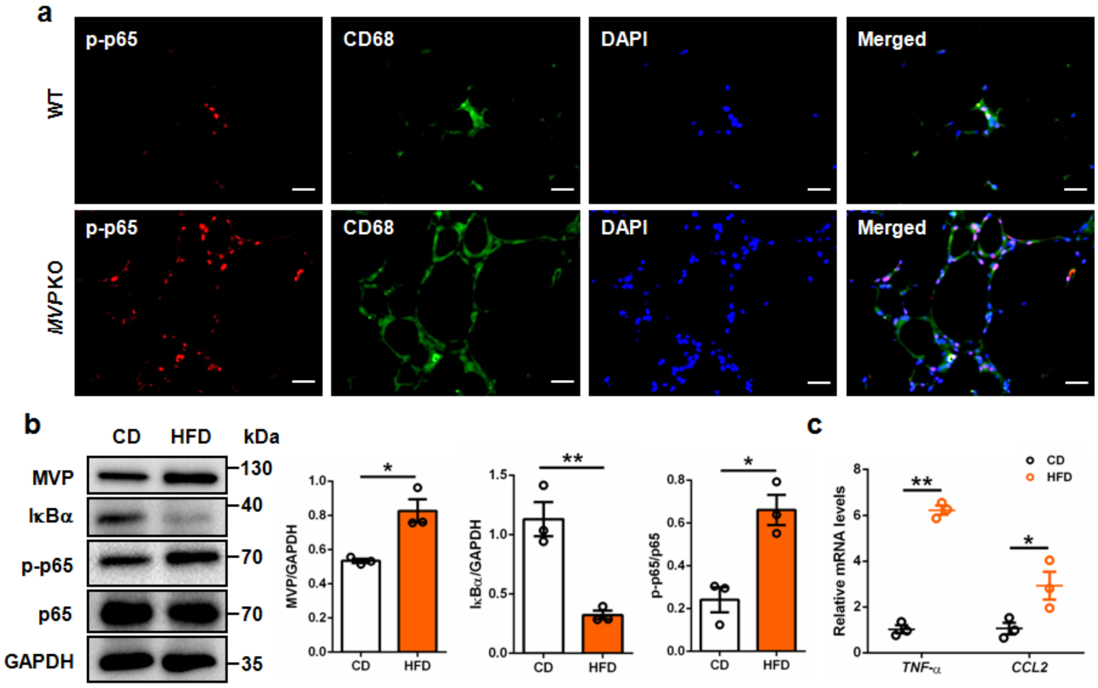

Supplementary Figure 8. (a) Representative immunofluorescence images of macrophage p-p65 staining with antibodies against p-p65 (red) and CD68 (green) in epiWAT of male WT and *MVP* KO mice fed a HFD for 12 weeks. Nuclei were stained with DAPI (blue). Scale bars, 50 μm. **(b)** Western blot analysis of MVP, IκBα, p-p65, and p65 in sorted F4/80^{+} macrophages isolated from CD- and HFD-fed male mice epiWAT SVFs (n = 3). **(c)** mRNA levels of *TNF-α* and *CCL2* in the isolated F4/80^{+} macrophages from CD- and HFD-fed male murine epiWAT SVFs (n = 3). Data are expressed as mean ± SEM. *P < 0.05 and **P < 0.01 by Student's *t* test.

Supplementary Figure 9.

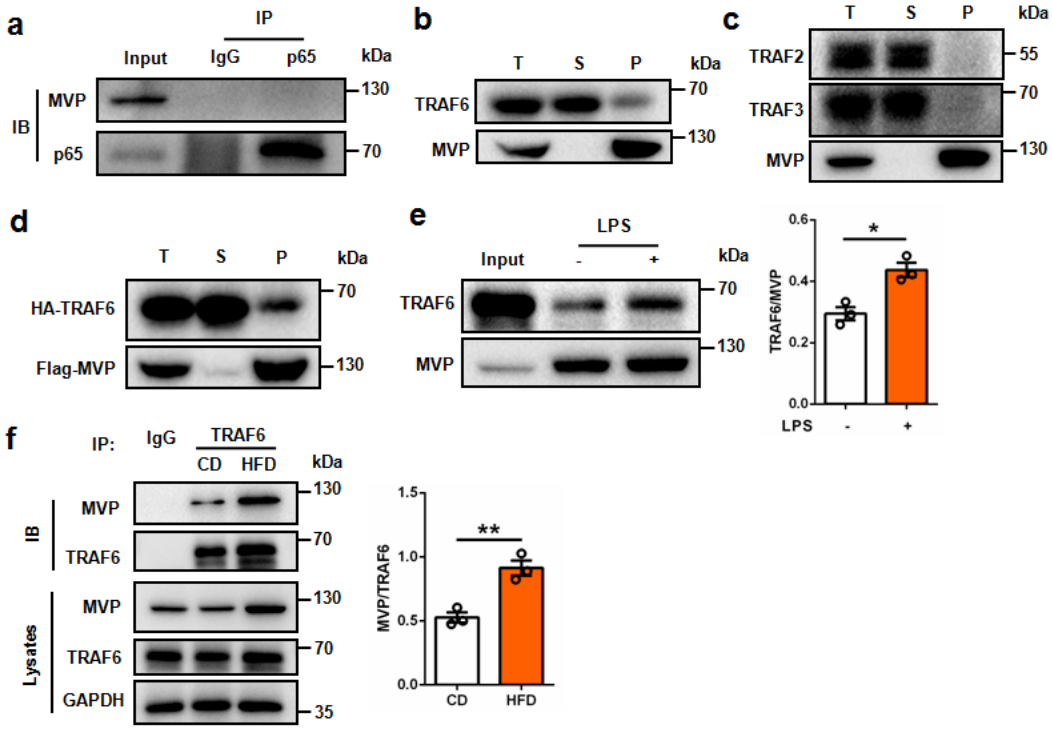

Supplementary Figure 9. (a) Co-IP and western blot analysis of endogenous MVP-p65 interaction in PMs. **(b-c)** BMDMs from CD-fed WT mice were cultured, harvested and used for cell fractionation by centrifugation at 100,000 g. Western blot analysis of MVP, TRAF6 (b), TRAF2 and TRAF3 (c) in total cell lysates (T), supernatants (S) and pellets (P). **(d)** HEK293T cells transfected by HA-TRAF6 and Flag-MVP were harvested and used for cell fractionation. Western blot analysis of HA-TRAF6 and Flag-MVP in T, S and P. **(e)** BMDMs from CD-fed WT mice were treated with or without LPS (100 ng ml^{-1}) treatment for 30 min, harvested and used for cell fractionation. Western blot analysis of MVP-TRAF6 complex formation in the pellets (n = 3). **(f)** Co-IP and western blot analysis of the interaction between MVP and TRAF6 in the epiWAT SVFs of CD- and HFD-fed male mice (n = 3). Representative results from three independent experiments are shown. Data are expressed as mean ± SEM. *P < 0.05 and **P < 0.01 by Student's *t* test.

Supplementary Figure 10.

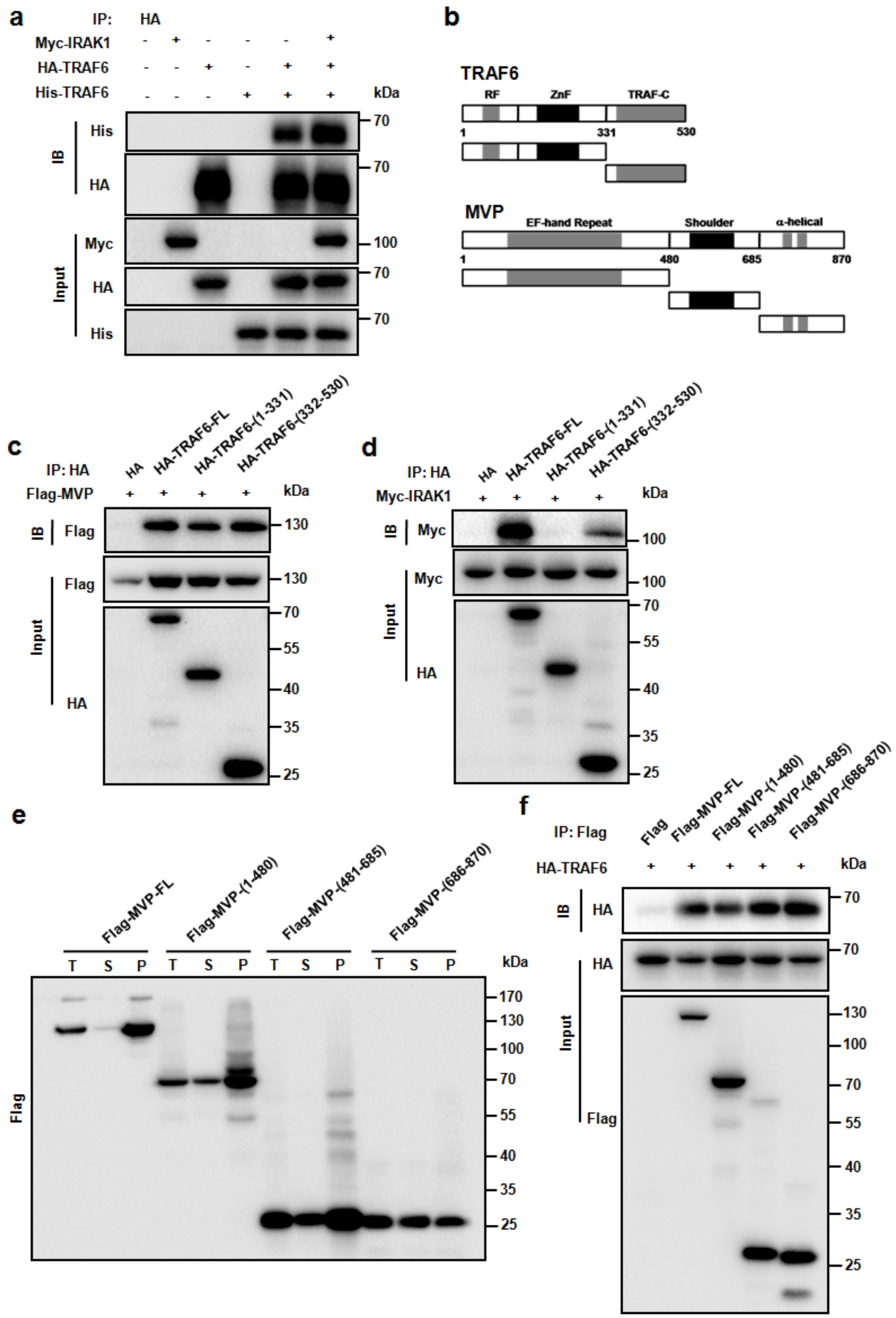

Supplementary Figure 10. (a) Co-IP and western blot analysis of the effect of IRAK1 on the oligomerization of TRAF6 in HEK293T cells. **(b)** Schematic of

full-length and truncated TRAF6 and MVP. **(c)** Co-IP and western blot analysis of the interaction between MVP and full-length and truncated TRAF6 in HEK293T cells. **(d)** Co-IP and western blot analysis of the interaction between IRAK1, full-length and truncated TRAF6 in HEK293T cells. **(e)** HEK293T cells transfected by Flag-tagged full length and truncated MVPs were harvested and used for cell fractionation. The proteins of total cell lysates (T), supernatants (S) and pellets (P) were detected by western blot with Flag antibody. **(f)** Co-IP and western blot analysis of the interaction between full-length, truncated MVPs and TRAF6 in HEK293T cells.

Supplementary Figure 11.

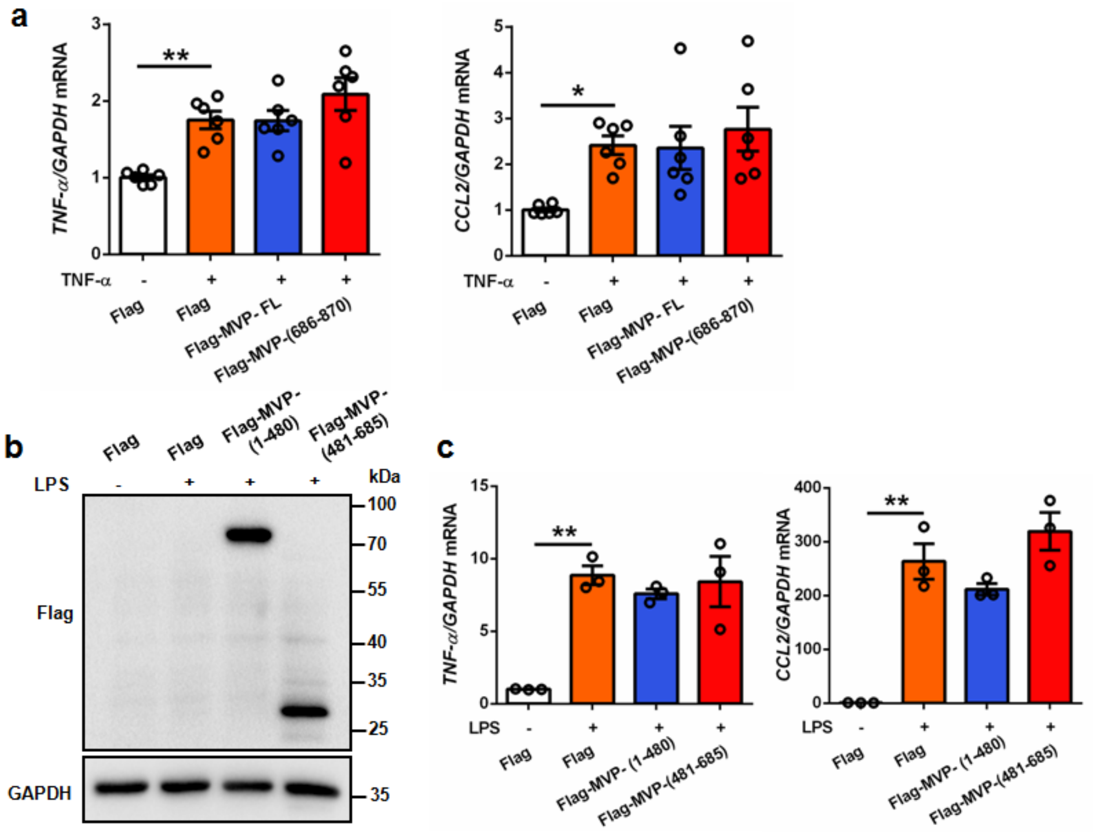

Supplementary Figure 11. (a) RAW264.7 cells were transfected by lenti-viruses expressing control Flag, Flag-MVP-FL and Flag-MVP-(686-870). After treated by TNF-α (10 ng ml^{-1}) for 1h, expression of *TNF-α* and *CCL2* in cells were measured by RT-qPCR (n = 6). **(b-c)** RAW264.7 cells were transfected by lenti-viruses expressing control Flag, Flag-MVP-(1-480) and Flag-MVP-(481-685). Western blot analysis of Flag-MVP-(1-480) and Flag-MVP-(481-685) in RAW264.7 cells (b). RAW264.7 cells were treated by LPS (100 ng ml^{-1}) for 12 h. Expression of *TNF-α* and *CCL2* in cells were measured by RT-qPCR (n = 3) (c). Representative results from three independent experiments are shown. Data are expressed as mean ± SEM. *P < 0.05 and **P < 0.01 by ANOVA with post hoc test.

Supplementary Figure 12.

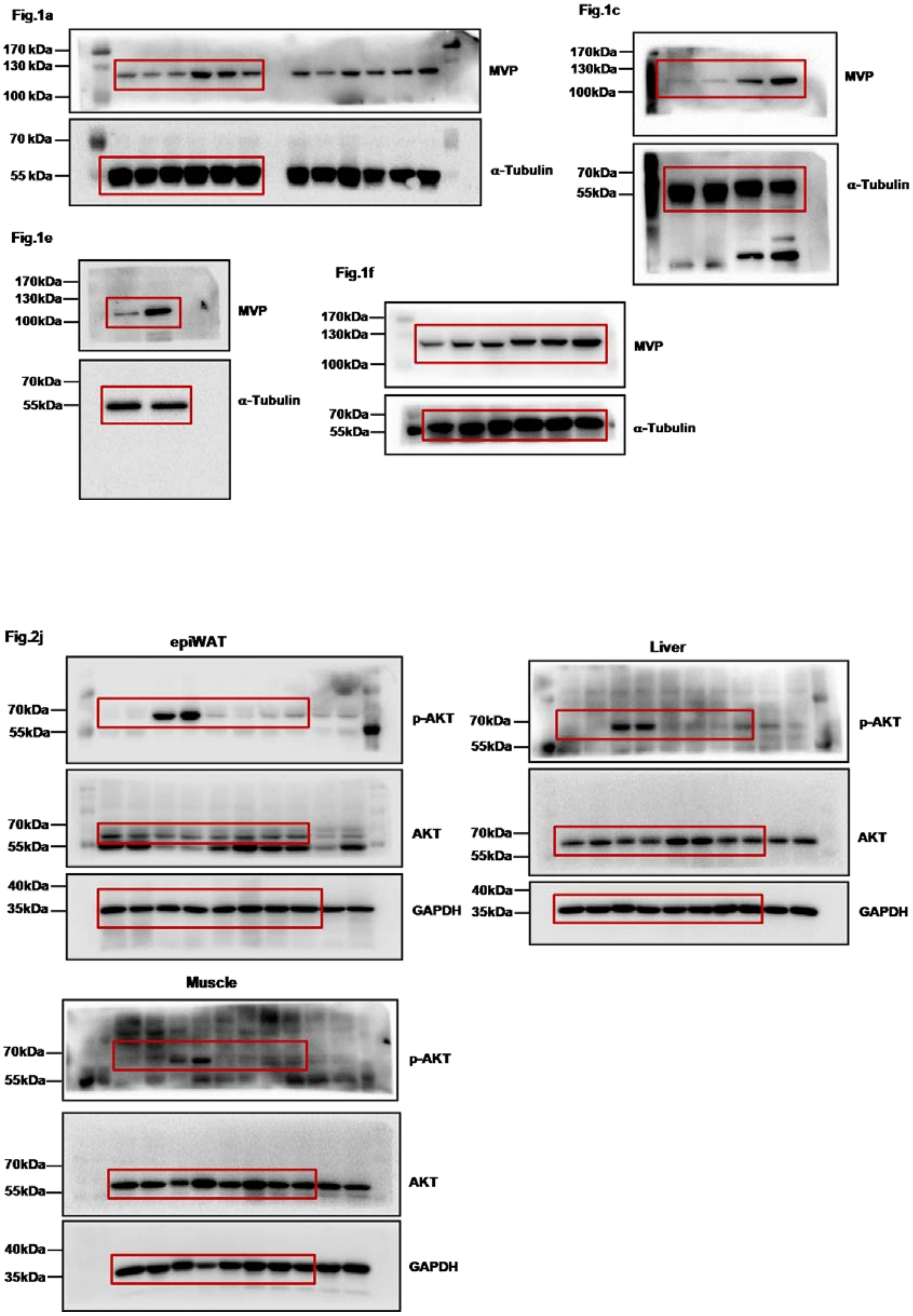

Fig.3a

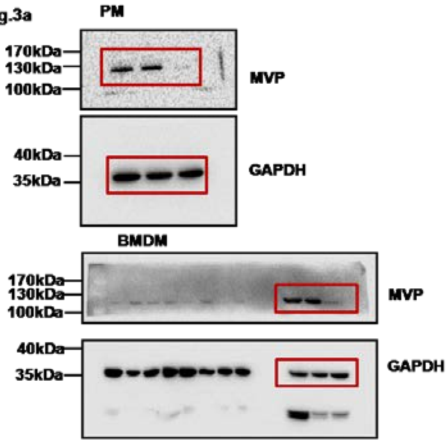

Fig.3k

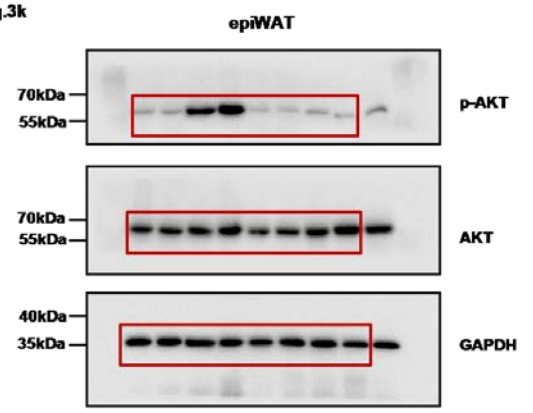

Fig.3k

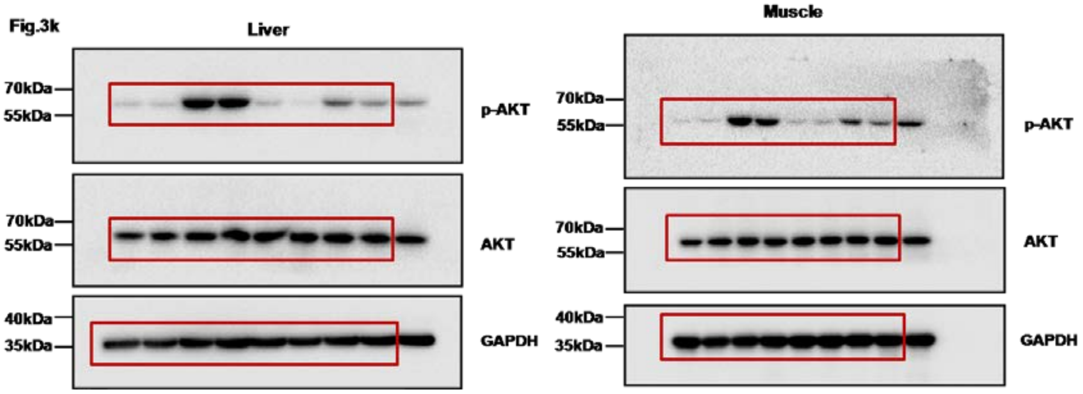

Fig.6c

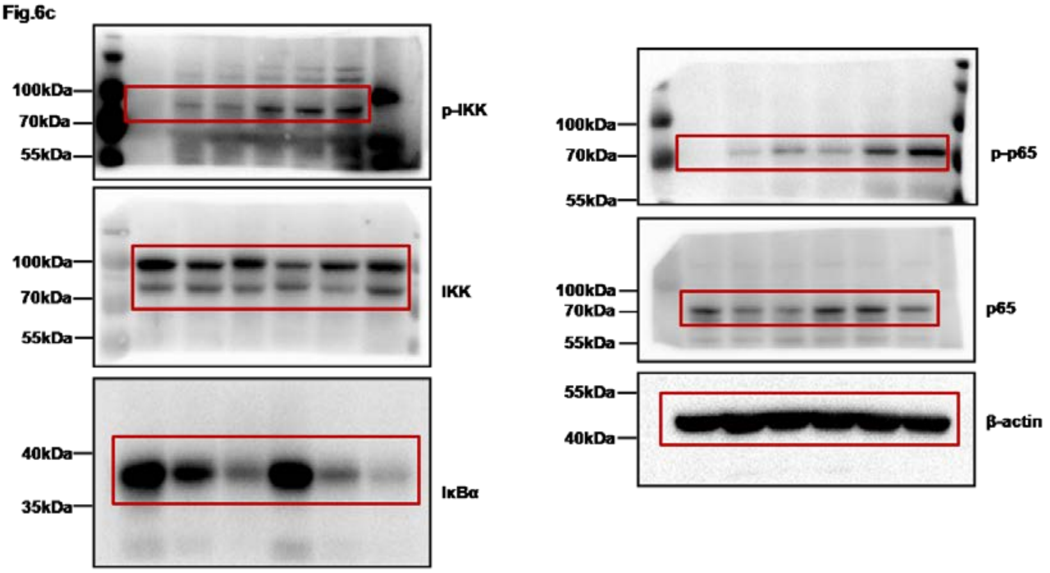

Fig.6e

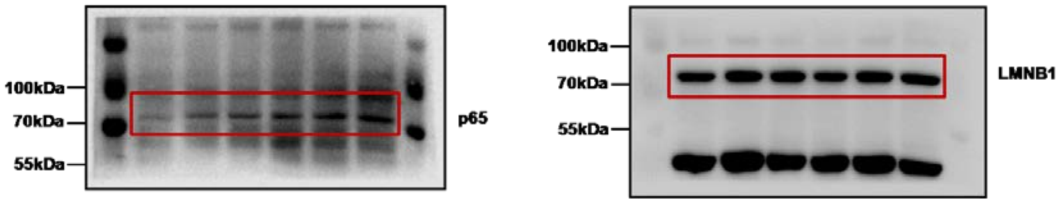

Fig.6j

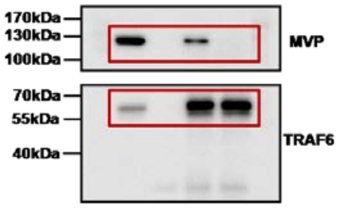

Fig.6k

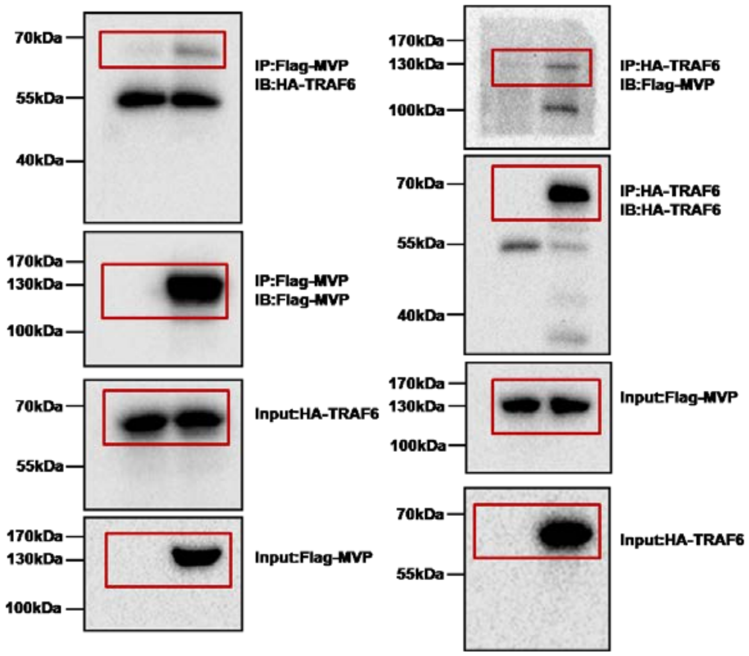

Fig.6l

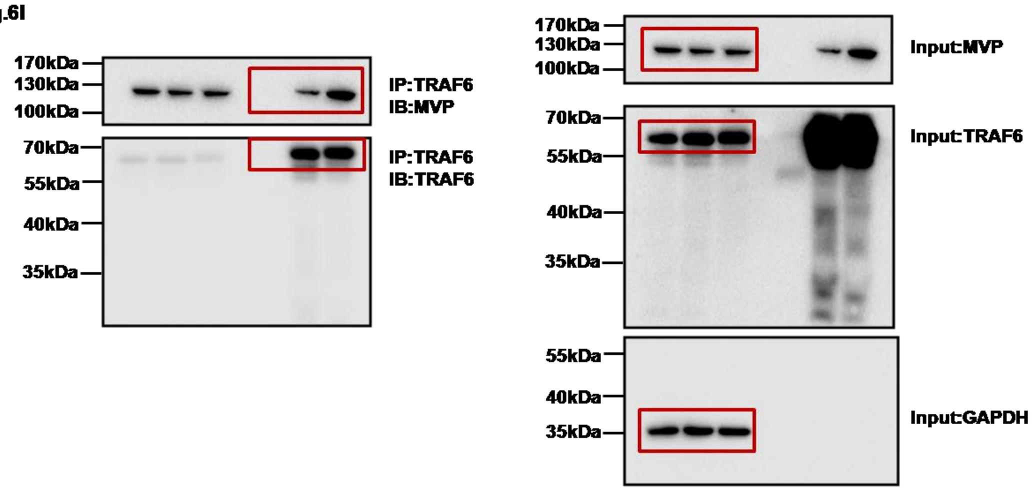

Fig.7a

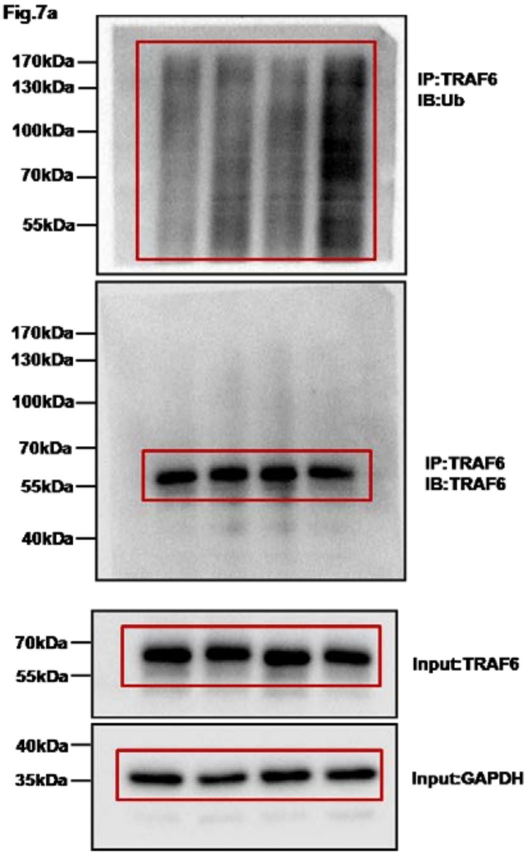

Fig.7b

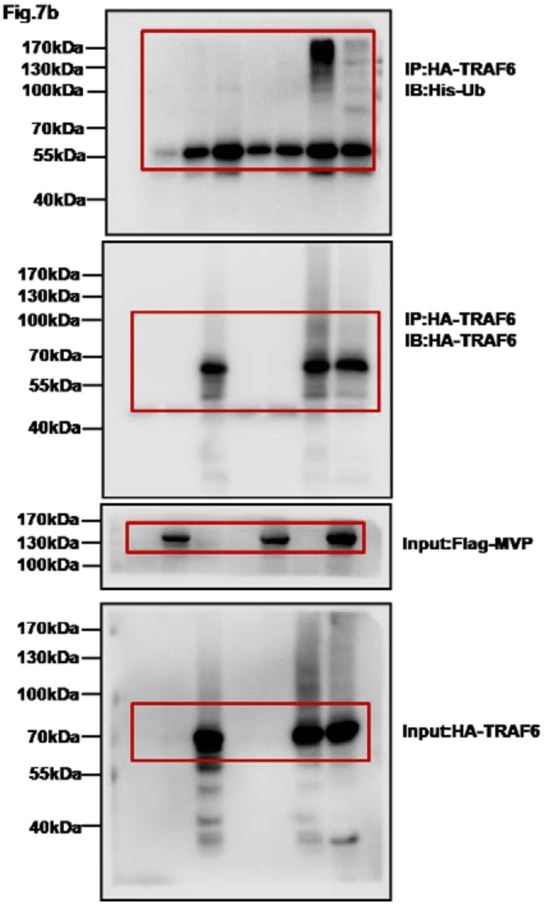

Fig.7c

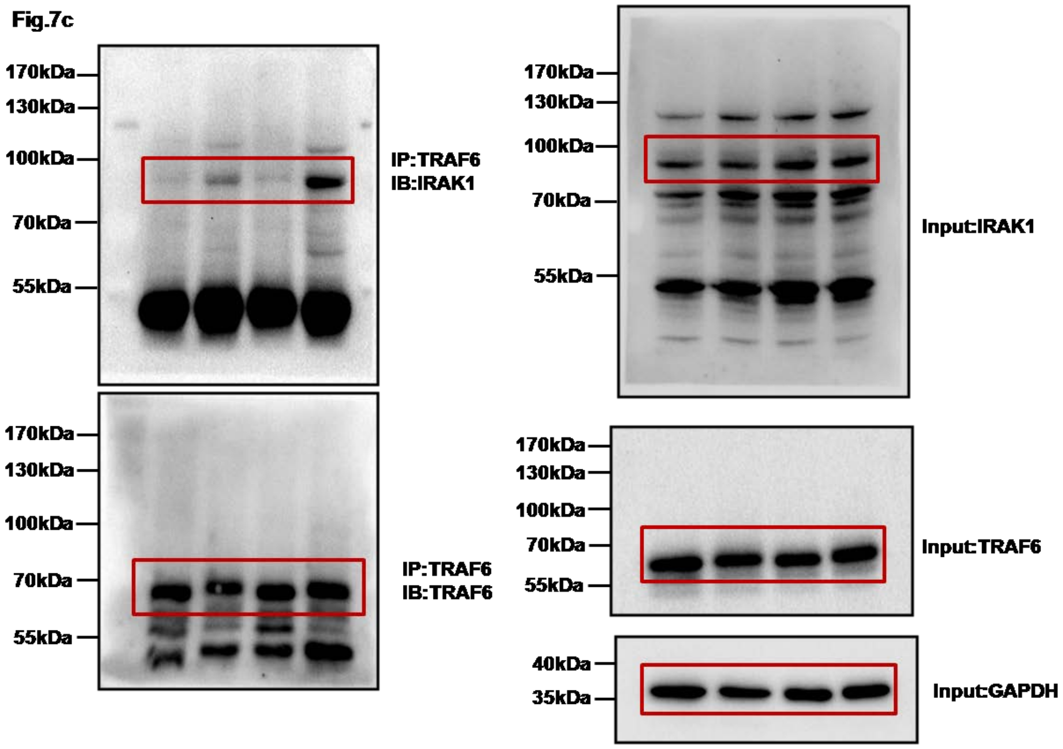

Fig.7d

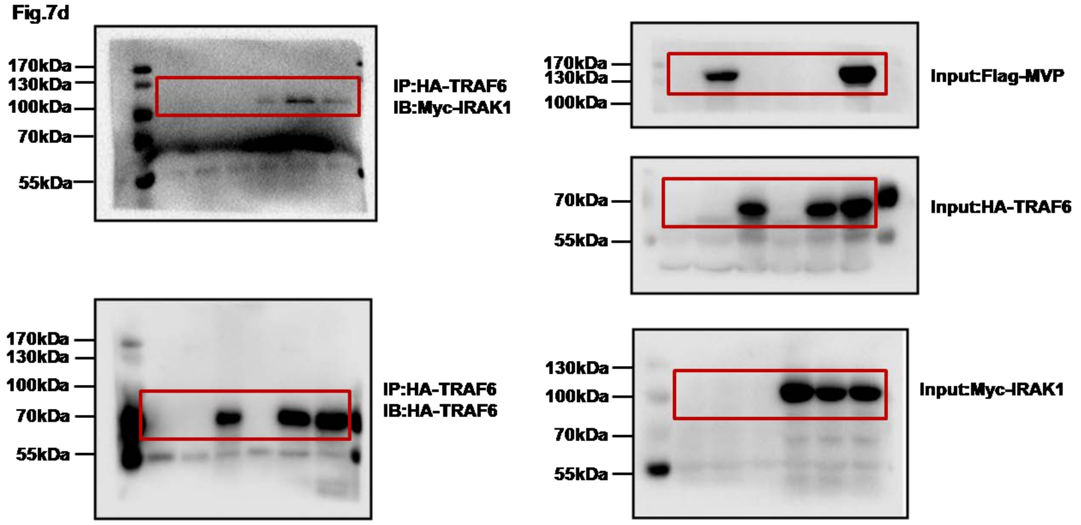

Fig.7e

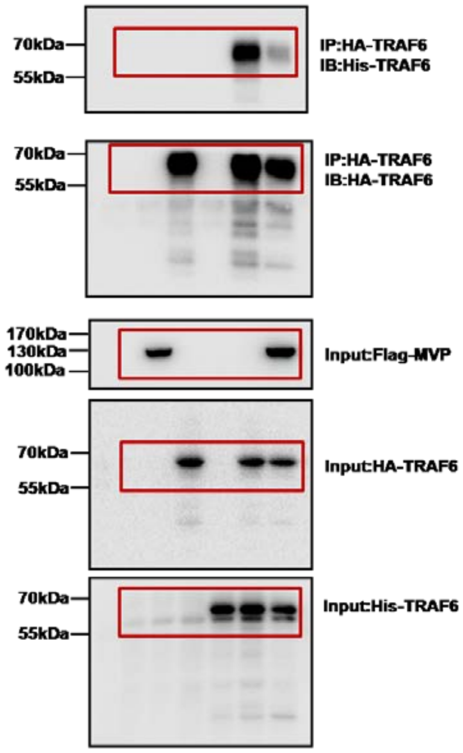

Fig.7f

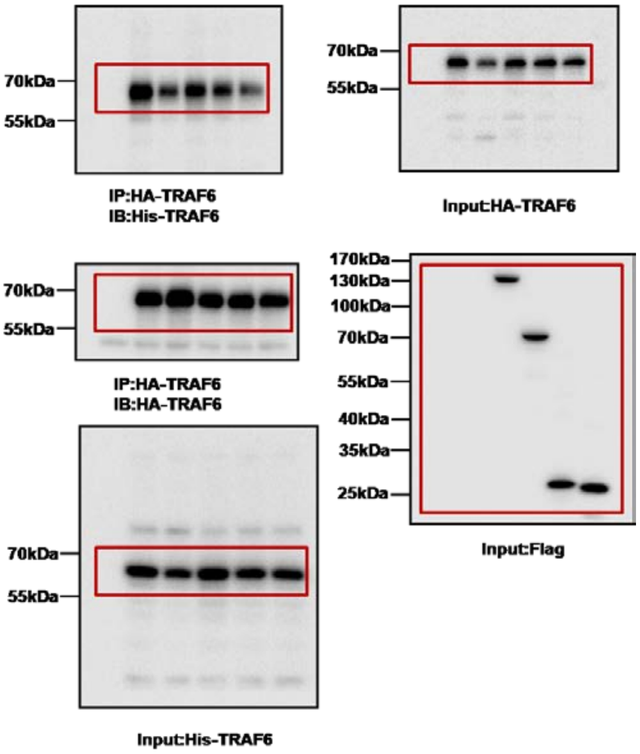

Fig.7g

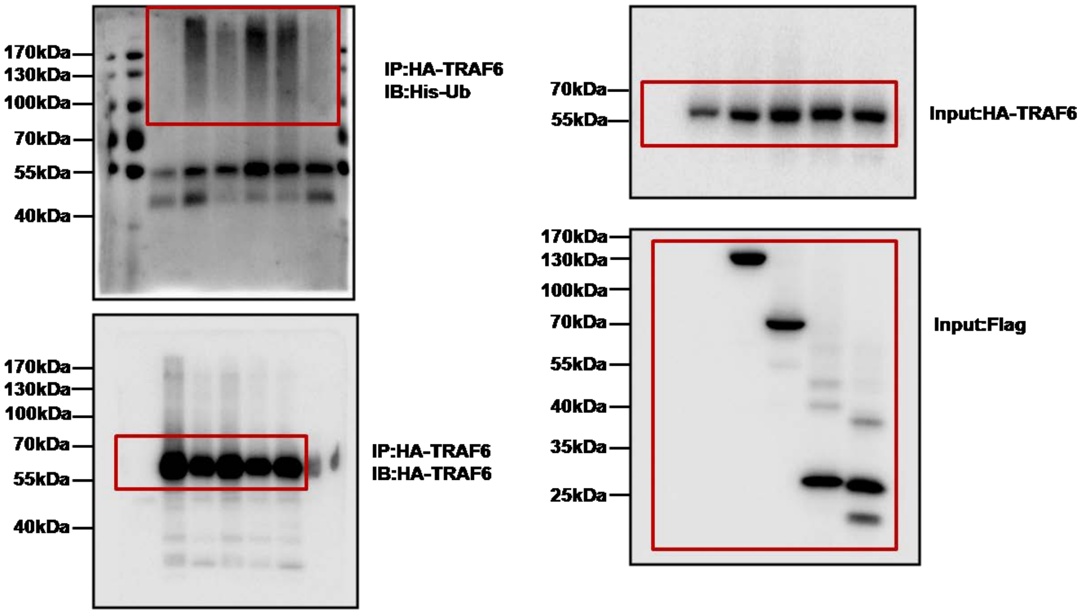

Fig.7h

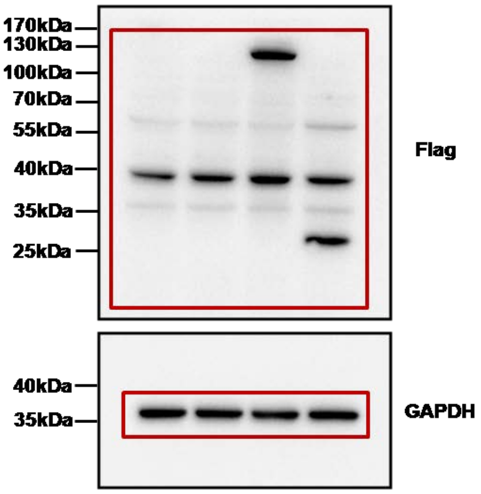

Fig.7k

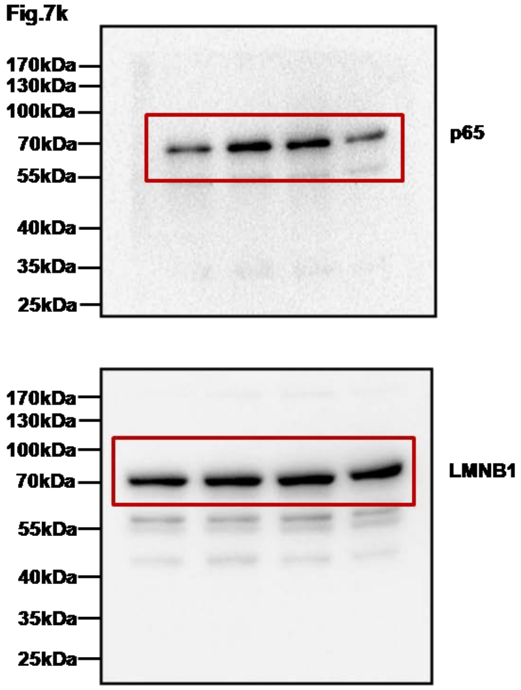

Fig.S1a

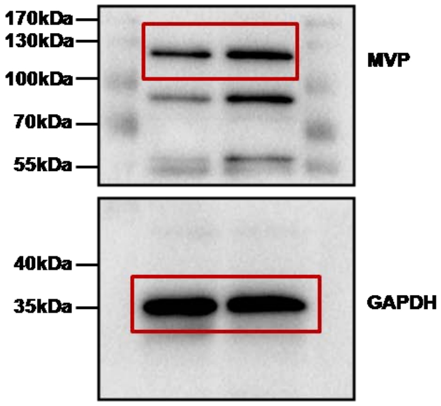

Fig.S1b

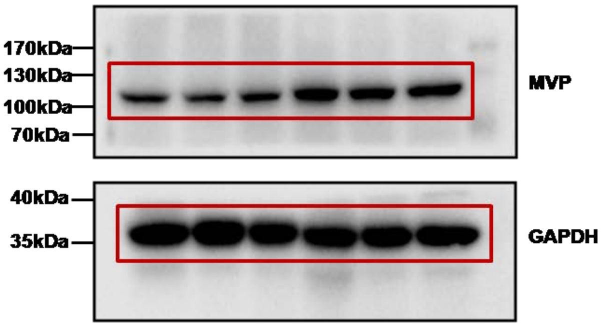

Fig.S4c

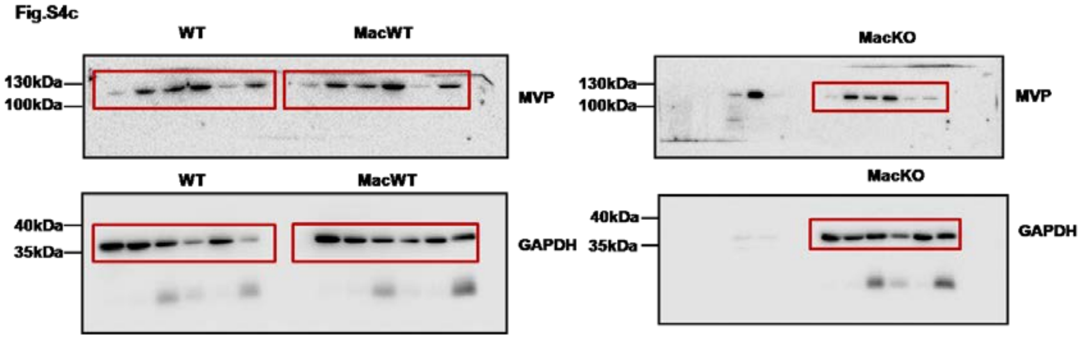

Fig.S6a

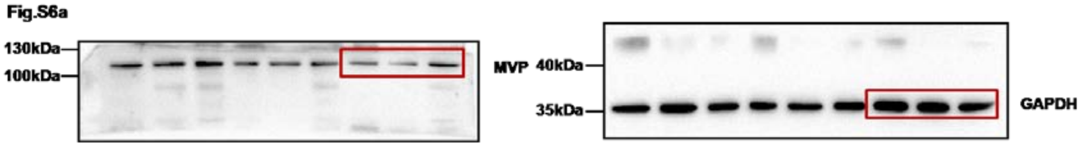

Fig.S8b

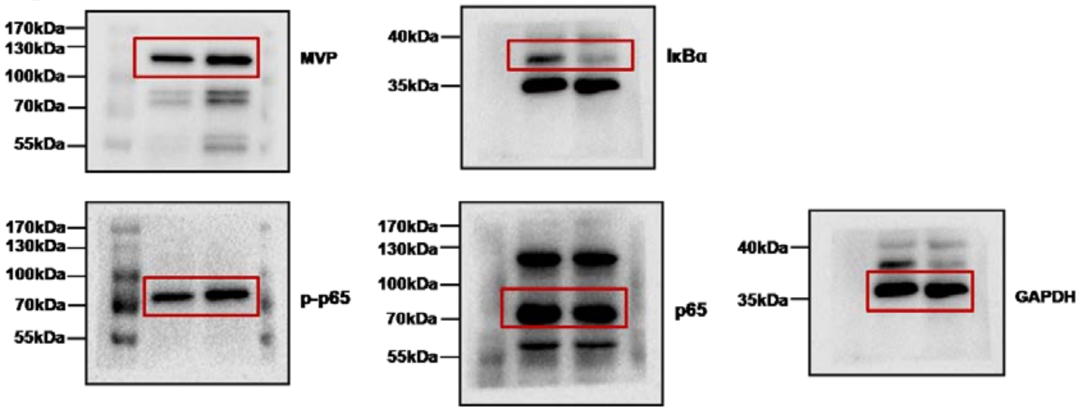

Fig.S9a

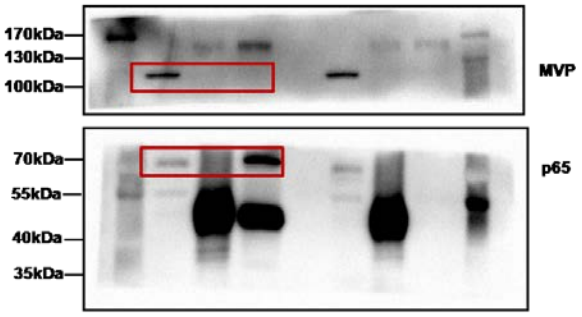

Fig.S9b

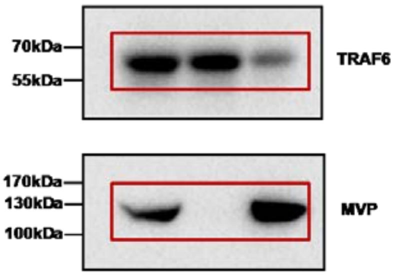

Fig.S9c

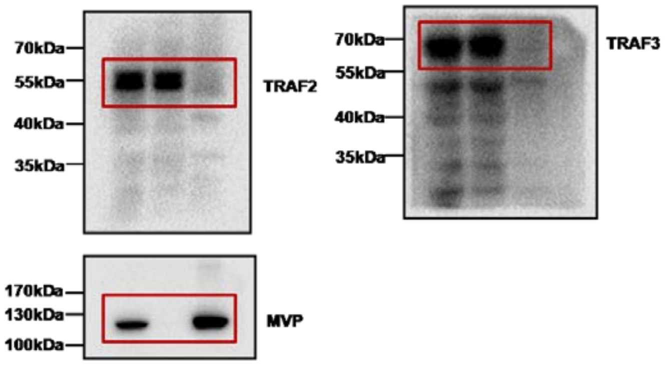

Fig.S9d

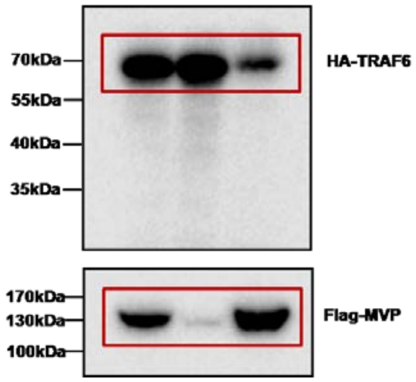

Fig.S9e

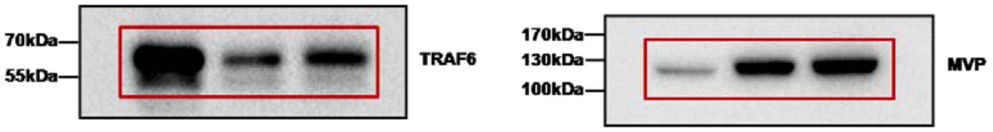

Fig.S9f

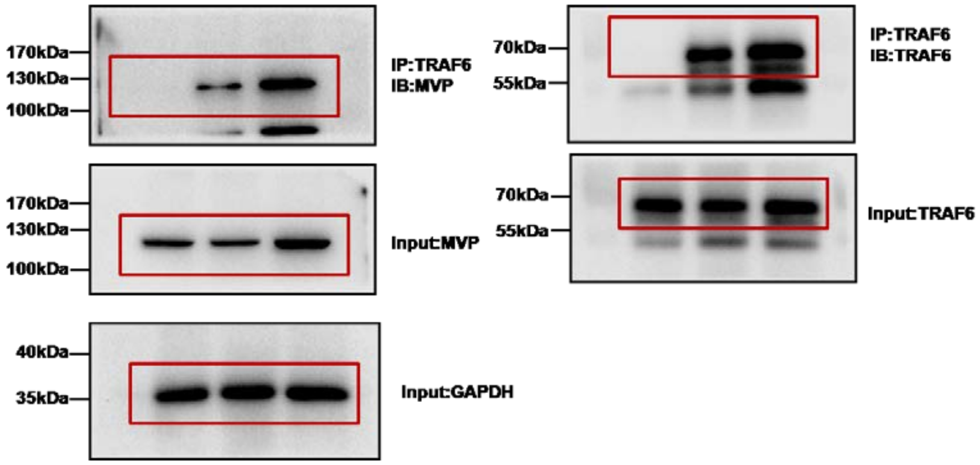

Fig.S10a

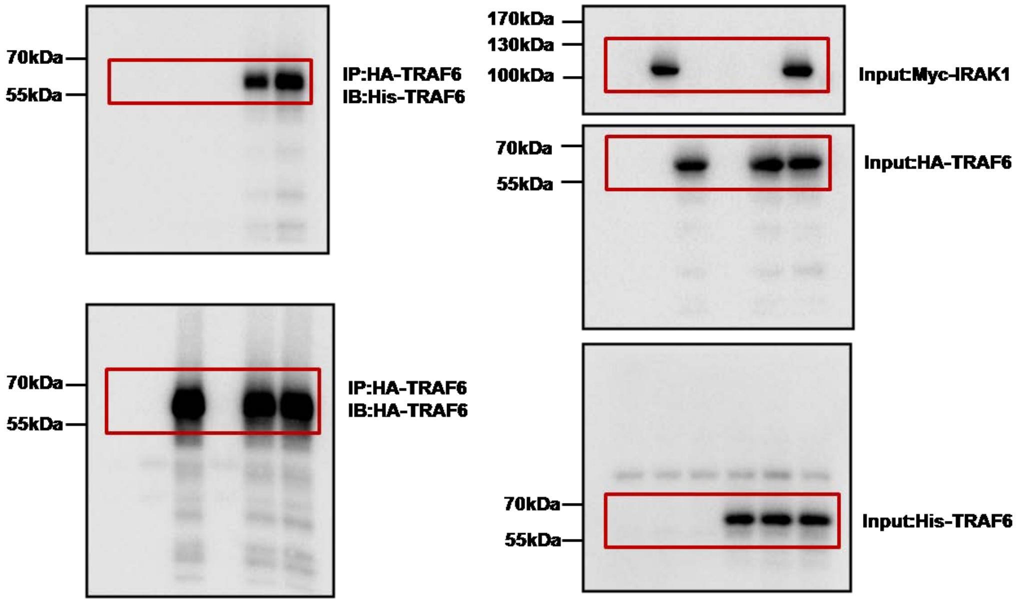

Fig.S10c

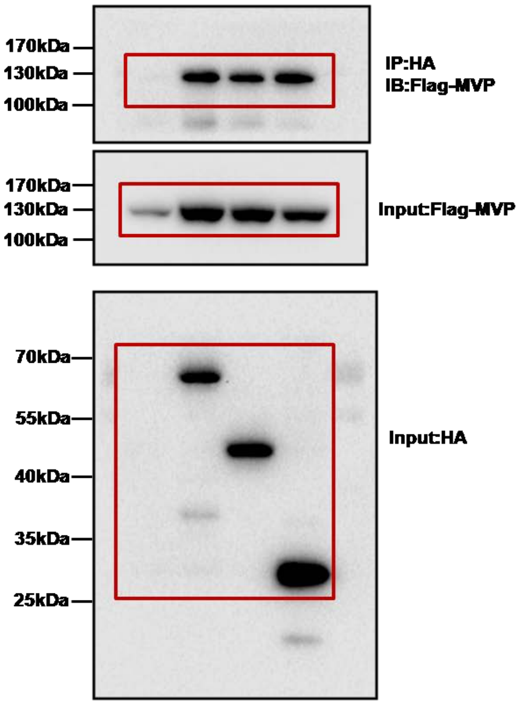

Fig.S10d

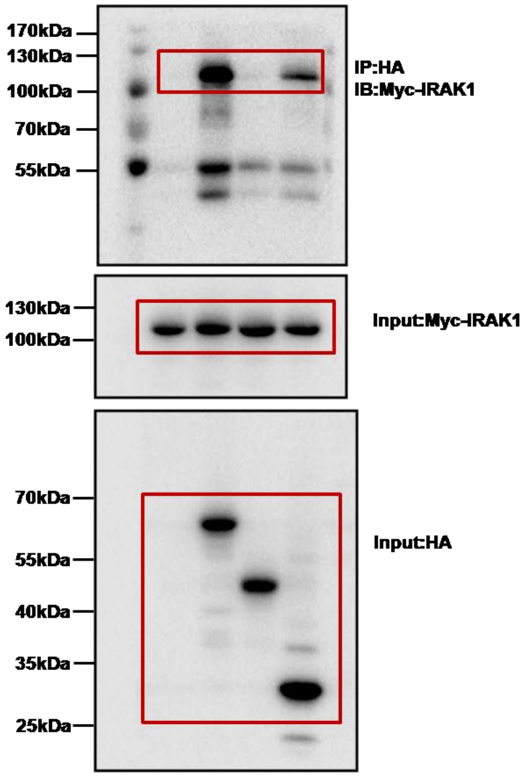

Fig.S10e

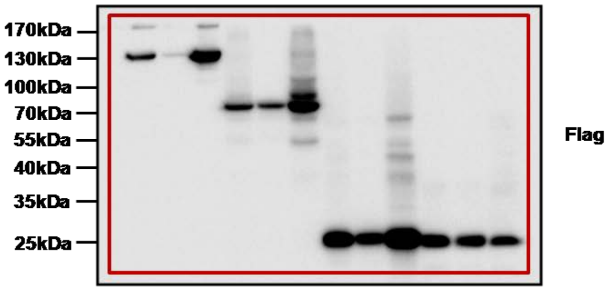

Fig.S10f

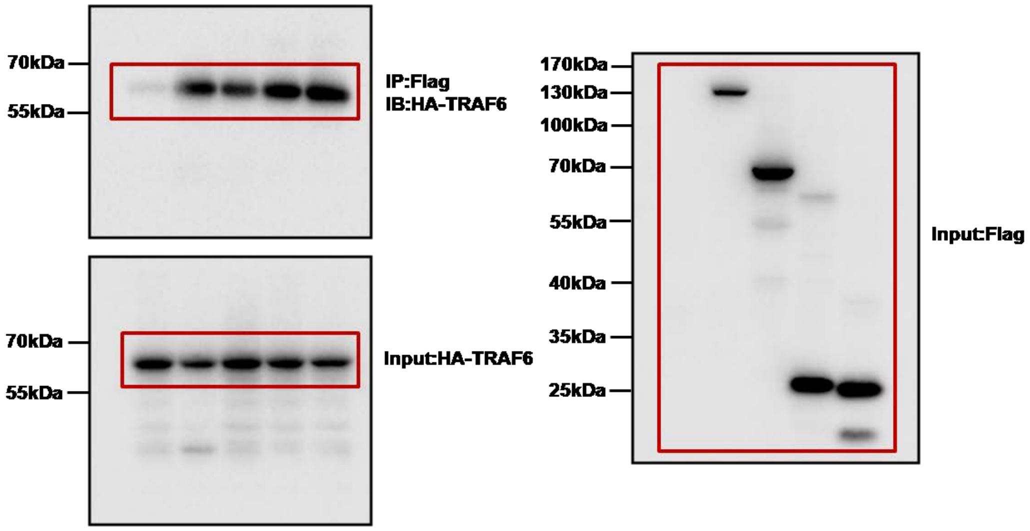

Fig.S11a

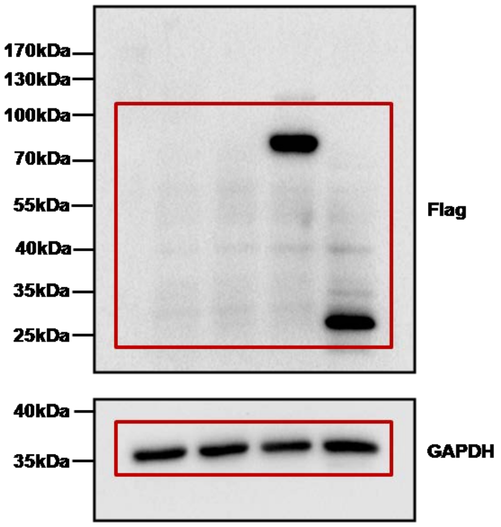

Supplementary Table 1. Primer pairs used for RT-qPCR

Gene	Forward primer (5'-3')	Reverse primer (5'-3')
h-MVP	GTTTGATGTCACAGGGCAAGTTCG	CCTTTAGATGGAGGGCAGTGTTGG
h-GAPDH	GGAAGGTGAAGGTCGGAGTCAACG	CTCGCTCCTGGAAGATGGTGATGG
GAPDH	AGGTCGGTGTGAACGGATTTG	TGTAGACCATGTAGTTGAGGTCA
LEP	GACACCAAAACCCTCAT	CAGTGTCTGGTCCATCT
ADIPOQ	GCACTGGCAAGTTCTACTGCAA	GTAGGTGAAGAGAACGGCCTTGT
FASN	CTGCGGAAACTTCAGGAAATG	GGTTCGGAATGCTATCCAGG
SCD1	TCTTCCTTATCATTGCCAACACCA	GCGTTGAGCACCAGAGTGTATCG
SREBP-1C	GCGCTACCGGTCTTCTATCA	TGCTGCCAAAAGACAAGGG
PPARγ	ATTCTGGCCCACCAACTTCGG	TGGAAGCCTGATGCTTTATCCCCA
CD36	ATGGGCTGTGATCGGAACTG	GTCTTCCCAATAAGCATGTCTCC
CPT-1α	CTCAGTGGGAGCGACTCTTCA	GGCCTCTGTGGTACACGACAA
LCAD	GCGAAATACTGGGCATCTGAA	TCCGTGGAGTTGCACACATT
MCAD	GACATTTGGAAAGCTGCTAGTG	TCACGAGCTATGATCAGCCTCTG
PDK4	TTCACACCTTCACCACATGC	AAAGGGCGGTTTTCTTGATG
UCP2	GCTGGTGGTGGTCGGAGATA	ACTGGCCCAAGGCAGAGTT
TNF-α	GTTCTATGGCCCAGACCCTCAC	GGCACCACTAGTTGGTTGTCTTTG
IL-6	CCACTTCACAAGTCGGAGGCTTA	GCAAGTGCATCATCGTTGTTCATAC
IL-1β	TCCAGGATGAGGACATGAGCAC	GAACGTCACACACCAGCAGGTTA
CCL2	AGCAGCAGGTGTCCCAAAGA	GTGCTGAAGACCTTAGGGCAGA
CCL3	CATGACACTCTGCAACCAAGTCTTC	GAGCAAAGGCTGCTGGTTTCA
CCL5	GGAGTATTTCTACACCAGCAGCAAG	GGCTAGGACTAGAGCAAGCAATGAC
CCL7	CAATGCATCCACATGCTGCTA	GACCCACTTCTGATGGGCTTC
CXCL1	TGCACCCAAACCGAAGTC	GTCAGAAGCCAGCGTTCACC
