# Supplementary material for: Major vault protein suppresses obesity and atherosclerosis through inhibiting IKK–NF-κB signaling mediated inflammation
Source: Nat Commun. 2019 Apr 17;10:1801. doi: 10.1038/s41467-019-09588-x (PMC6470148; doi:10.1038/s41467-019-09588-x)
Supplement: Supplementary file 3 — Reporting Summary [file 41467_2019_9588_MOESM3_ESM.pdf]

nature research

Corresponding author(s): Qi Chen, Jingjing Ben

Last updated by author(s): Jan 30, 2019

Reporting Summary

Nature Research wishes to improve the reproducibility of the work that we publish. This form provides structure for consistency and transparency in reporting. For further information on Nature Research policies, see Authors & Referees and the Editorial Policy Checklist.

Statistics

For all statistical analyses, confirm that the following items are present in the figure legend, table legend, main text, or Methods section.

n/a	Confirmed	
☐	☒	The exact sample size (*n*) for each experimental group/condition, given as a discrete number and unit of measurement
☐	☒	A statement on whether measurements were taken from distinct samples or whether the same sample was measured repeatedly
☐	☒	The statistical test(s) used AND whether they are one- or two-sided<br>*Only common tests should be described solely by name; describe more complex techniques in the Methods section.*
☒	☐	A description of all covariates tested
☐	☒	A description of any assumptions or corrections, such as tests of normality and adjustment for multiple comparisons
☐	☒	A full description of the statistical parameters including central tendency (e.g. means) or other basic estimates (e.g. regression coefficient) AND variation (e.g. standard deviation) or associated estimates of uncertainty (e.g. confidence intervals)
☐	☒	For null hypothesis testing, the test statistic (e.g. *F*, *t*, *r*) with confidence intervals, effect sizes, degrees of freedom and *P* value noted<br>*Give P values as exact values whenever suitable.*
☒	☐	For Bayesian analysis, information on the choice of priors and Markov chain Monte Carlo settings
☒	☐	For hierarchical and complex designs, identification of the appropriate level for tests and full reporting of outcomes
☐	☒	Estimates of effect sizes (e.g. Cohen's *d*, Pearson's *r*), indicating how they were calculated

Our web collection on statistics for biologists contains articles on many of the points above.

Software and code

Policy information about availability of computer code

Data collection	BD FACSuite software was used for cytofluorimetric data. Zeiss ZEN 2012 was used for image collection.
Data analysis	GraphPad Prism (v6); FlowJo (v10); ImageJ (c1.48); ImagePro Plus (v6)

For manuscripts utilizing custom algorithms or software that are central to the research but not yet described in published literature, software must be made available to editors/reviewers. We strongly encourage code deposition in a community repository (e.g. GitHub). See the Nature Research guidelines for submitting code & software for further information.

Data

Policy information about availability of data

All manuscripts must include a data availability statement. This statement should provide the following information, where applicable:

- Accession codes, unique identifiers, or web links for publicly available datasets
- A list of figures that have associated raw data
- A description of any restrictions on data availability

All data supporting the findings of this study are available within the main manuscript and the supplementary files, or from the corresponding author upon reasonable request.

Field-specific reporting

Please select the one below that is the best fit for your research. If you are not sure, read the appropriate sections before making your selection.

☒ Life sciences ☐ Behavioural & social sciences ☐ Ecological, evolutionary & environmental sciences

Life sciences study design

All studies must disclose on these points even when the disclosure is negative.

Sample size	We used standard sample sizes reported in the literature previously in mouse studies. The sample size of animal experiments according to similar studies in the field was chose and at least 5 mice per group was used. The number of the independent experiments for cell and biological experiments was indicated in each figure legend.
Data exclusions	No data were excluded from the analyses.
Replication	All experiments were performed independently multiple times using biologically independent replicates.
Randomization	Mice were randomly assigned to groups.
Blinding	The investigators were blinded to the identities of the samples. We collected and analyzed the compared samples under the same conditions.

Reporting for specific materials, systems and methods

We require information from authors about some types of materials, experimental systems and methods used in many studies. Here, indicate whether each material, system or method listed is relevant to your study. If you are not sure if a list item applies to your research, read the appropriate section before selecting a response.

Materials & experimental systems

n/a	Involved in the study
☐	☒ Antibodies
☐	☒ Eukaryotic cell lines
☒	☐ Palaeontology
☐	☒ Animals and other organisms
☐	☒ Human research participants
☒	☐ Clinical data

Methods

n/a	Involved in the study
☒	☐ ChIP-seq
☐	☒ Flow cytometry
☒	☐ MRI-based neuroimaging

Antibodies

Antibodies used	Antibodies used in this study include: MVP (Santa Cruz, sc-23916), MVP (Santa Cruz, sc-18701), phospho-AKT (Cell Signaling, 9271), AKT (Cell Signaling, 9272), phospho-IKKα/β (Cell Signaling, 2697), IKKα/β (Santa Cruz Biotechnology, sc-7607), IκBα (Cell Signaling, 4814), phospho-p65 (Cell Signaling, 3033), p65 (Cell Signaling, 8242), TRAF6 (BioLegend, 654502), TRAF6 (Thermo Fisher Scientific, 38-0900), TRAF6 (Absin, abs115194), Flag (Sigma-Aldrich, F1804), HA (Roche, 11867423001), HA (Thermo Fisher Scientific, 26183), TRAF2 (Cell Signaling, 4724), TRAF3 (Santa Cruz, sc-6933), Ub (Milipore, MAB1510), IRAK1 (Santa Cruz, sc-5288), Myc (Cell Signaling, 2278), His (Cell Signaling, 12698), CD68 (Bio-Rad, MCA1957), Perilipin (Cell Signaling, 9349), α-Tubulin (Protein Tech, 11224-1-AP), GAPDH (Kangchen Tech, KC-5G4), β-actin (Santa Cruz, sc-47778), Lamin B1 (Protein Tech, 66095-1-Ig), BV421 anti-F4/80 (BD Biosciences, 565411), FITC anti-CD11b (BD Biosciences, 553310), PE anti-F4/80 (R&D systems, FAB5580P)
Validation	The antibodies are from commercial sources and have been validated by the vendors.

Eukaryotic cell lines

Policy information about cell lines

Cell line source(s)	HEK293T and RAW264.7 were obtained from the American Type Culture Collection (ATCC).
Authentication	All of the cell lines were authenticated by short tandem repeat (STR)-profiling.
Mycoplasma contamination	All cell lines used in this study tested negative for mycoplasma before their use.
Commonly misidentified lines (See ICLAC register)	No commonly misidentified cell lines were used in this study.

Animals and other organisms

Policy information about studies involving animals; ARRIVE guidelines recommended for reporting animal research

Laboratory animals	All animal protocols were approved by the Institutional Animal Care and Use Committee of Nanjing Medical University. Mice

Laboratory animals	were housed at 22 °C - 24 °C under standard light conditions (12 h light/dark cycle) and were allowed free access to water and food. Details are described in the methods section.
Wild animals	The study did not involve wild animals.
Field-collected samples	The study did not involve field-collected samples.
Ethics oversight	Institutional Animal Care and Use Committee of Nanjing Medical University

Note that full information on the approval of the study protocol must also be provided in the manuscript.

Human research participants

Policy information about studies involving human research participants

Population characteristics	Human tissue biopsies from visceral adipose tissue were obtained from the Bayi Clinical Medicine School of Nanjing Medical University during surgery. All subjects provided their written informed consent. All procedures that involved human samples were approved by the Ethics Committee of Bayi Clinical Medicine School of Nanjing Medical University.
Recruitment	These patients were recruited while in hospital awaiting surgery. They were divided by BMI, while 18.5 ≤ BMI < 24 as normal weight, 24 ≤ BMI < 28 as overweight and BMI ≥ 28 as obese. The gender and age was not considered in the analysis and only BMI. There were no obvious study selection biases that would have influenced the results except for the sample size, thereby increasing variability.
Ethics oversight	Ethics Committee of Bayi Clinical Medicine School of Nanjing Medical University

Note that full information on the approval of the study protocol must also be provided in the manuscript.

Flow Cytometry

Plots

Confirm that:

☒ The axis labels state the marker and fluorochrome used (e.g. CD4-FITC).

☒ The axis scales are clearly visible. Include numbers along axes only for bottom left plot of group (a 'group' is an analysis of identical markers).

☒ All plots are contour plots with outliers or pseudocolor plots.

☒ A numerical value for number of cells or percentage (with statistics) is provided.

Methodology

Sample preparation	To isolate SVFs, adipose tissues were chopped finely and digested using collagenase type II (1.5 mg/ml, Sigma) at 37 °C for 40 min. After passing cells through a 200 um cell strainer and centrifugation at 1,000 g for 10 min, the pellet containing the SVFs was then incubated with red blood cell lysis buffer. SVFs were resuspended in PBS supplemented with 1 % FBS. Single cell suspensions were then stained for cell-surface markers with flourochrome-conjugated antibodies in PBS containing 1 % FBS in the dark for 30 min at room temperature. After the staining, the cells were washed and resuspended in 400uL PBS , and then acquired with FACS VERSE machine or sorted with FACS Aria II machine.
Instrument	FACS Aria II, FACS Verse
Software	All events were acquired using BD FACSDiva and FACSuite software and data were analyzed with FlowJo v10 software.
Cell population abundance	Sorted F4/80 positive SVFs were confirmed to be >90% following each sorting.
Gating strategy	Cells were gated by FSC-A x SSC-A to exclude debris and then by FSC-A x FSC-W following SSC-A x SSC-W to exclude cell doublets. For sorted, macrophages were identified as F4/80+ cells from SVFs of mice. For regular flow, macrophages were identified as F4/80+CD11b+ cells from SVFs of mice.

☐ Tick this box to confirm that a figure exemplifying the gating strategy is provided in the Supplementary Information.
